# Exclusive breastfeeding and maternal employment among mothers of infants from three to five months old in the Fafan zone, Somali regional state of Ethiopia: a comparative cross-sectional study

**DOI:** 10.1186/s12889-019-7345-5

**Published:** 2019-07-29

**Authors:** Frew Tadesse, Yonas Alemayehu, Sisay Shine, Henok Asresahegn, Trhas Tadesse

**Affiliations:** 1 Department of Public Health Officer, College of Medicine and Health Sciences, Jigjiga University, Jigjiga, Ethiopia; 2 Department of Nursing, College of Medicine and Health Sciences, Jigjiga University, Jigjiga, Ethiopia; 3 Department of Social and Population Health, Yekatit 12 Hospital Medical College, Addis Ababa, Ethiopia

**Keywords:** Exclusive breastfeeding, Maternal employment, Somali, Ethiopia

## Abstract

**Background:**

Lack of exclusive breastfeeding is the most important risk factor for infant and young child morbidity and mortality. A better understanding of the factors that influence EBF is important in order to promote appropriate infant feeding practices. The return to work due to short maternity leave time may hinder employed mothers from breastfeeding their infants exclusively for the recommended six months duration.

**Methods:**

A community based comparative cross-sectional study was conducted from January to February 2016 in the Fafan zone, of the Somali Regional State, of Ethiopia. A total of 558 mothers with infants from ages 3–5 months, living in the five districts (Jigjiga city, Kebribeyah town, Aubere town, Bombas town and Babile) were included in the analysis. Logistic regression models were used to examine the effect of maternal employment on EBF practice.

**Results:**

This study has demonstrated a 24.8 and 82.9% prevalence of EBF practices among employed and unemployed mothers of index infants of 3–5 months respectively in the 24 h preceding the survey. Unemployed mothers were accounted for thusly: [Adjusted OR = 26.5; 95% CI (13.6, 51.6). Other adjustments included monthly income of 500–2000 birr [Adjusted OR = 2.7; 95% CI (1.4, 5.2)]; monthly income of 2001–3500 birr [Adjusted OR = 2.2; 95% CI (1.2, 4.0)]; timely initiation of breastfeeding [Adjusted OR = 2.6; 95% CI (1.4, 4.8)]; maternal education (secondary and higher) [Adjusted OR = 3.8; 95% CI (1.5, 9.5)]; having an index infant aged 3 months [Adjusted OR = 2.2; 95% CI (1.2, 4.1)], and having an index infant aged 4 months [Adjusted OR = 2.2; 95% CI (1.2, 3.8)] were found to be significantly associated with exclusive breastfeeding practice.

**Conclusion:**

Exclusive breastfeeding practices were very low among mothers employed in governmental and non-governmental organizations in the study area. Therefore, maternal employment may be hindering Exclusive breastfeeding practices. Thus, establishing breastfeeding-friendly working environment; and Information, Education and Communication programs should be provided, particularly for working mothers to promote exclusive breastfeeding practices.

## Background

As per the definition of World Health Organization (WHO), exclusive breastfeeding (EBF) is defined as an infant’s consumption of breast milk without supplementation of any other food or drink, not even water except for oral rehydration salt (ORS), vitamins, minerals, and medications. Infants should receive EBF for the first six months of life [1]. Lack of EBF is the most important risk factors for infant and young child morbidity and mortality including life-long impact like poor school performance, reduced productivity, and impaired intellectual development [2, 3]. To reduce infant and young child mortality, EBF has been identified as one of the major cost-effective interventions worldwide [4–6]. In developing countries like Ethiopia, appropriate practice of EBF can avert 13% of under-five mortality [6]. Despite its proven benefits, the proportion of exclusively breastfed children up to 6 months is lower than the optimal recommendations [7–11]. Studies conducted in different parts of the country [12–14] indicate that more than 80% of mothers are knowledgeable about EBF. Nevertheless, only 52% of children under age 6 months are exclusively breastfed, and the percentage of EBF declines with age from 70% in 0–1 months to 32% in 4–5 months. The median duration of EBF is low among the pastoralist and semi pastoralist communities of the country. In particular, the Somali region had the lowest median duration of EBF which is 0.5 months [10].

Sustainable Development Goal 2 (SDG 2) targets include ending hunger especially amongst the vulnerable and the poor and ending all forms of nutrition especially stunting and wasting of children under the age of five years [15]. Hence, a better understanding of the factors that influence EBF is necessary to promote appropriate infant feeding practices and attain SDG 2. In developing countries like Ethiopia, various maternal and child factors including maternal employment have been responsible for the low prevalence of EBF [7, 8, 14, 16–19]. In this study, being an employed mother has been hypothesized as one of the main barriers to EBF. Because of increasing urbanization and level of education, the proportion of employed women in Ethiopia has been increasing gradually [10]. Employed women in the formal and informal sectors face challenges combining work with breastfeeding. The return to work due to short maternity leave time may influence employed mothers not to start breastfeeding at all or discontinue EBF earlier than the recommended duration [20–23]. A research conducted in other part of the country [16] demonstrated that employed mothers were less likely to exclusively breastfeed their infant(s) than unemployed mothers. Although several studies have been conducted on the factors that are associated with EBF, there has been insufficient information regarding the effects of maternal employment in the formal sector on EBF practices in the semi-pastoralist community of the country specifically. Therefore, this study is intended to bridge this gap by assessing association maternal employment and EBF practices among mothers with infants between the ages of 3–5 months. The findings of this study will be crucial to the implementation interventions that speed up the government efforts in promoting EBF, and thereby decrease the rates and burden of infant morbidity and mortality.

## Methods

### Study setting and population

This study was conducted from January to February 2016 among mothers with infants between the ages of 3–5 months, living in the Fafan zone, of the Somali Regional State, of Ethiopia. The Fafan zone, which contains the capital of the Somali Regional state (Jigjiga), is a semi pastoralist community located at the Eastern border of the country. Here there are many rural and urban migrants that are attracted by trading and employment opportunities. Primary employers include governmental, and non-governmental organizations NGOs). These assertions are based on the 2007 Census conducted by Central Statistical Agency of Ethiopia. This zone has a total population of 967,652, of which 526,398 are men and 441,254 women. About 12% of the 203,588 urban inhabitants were pastoralists [24].

The study participants were both employed, and unemployed mothers with infants from ages 3–5 months, living in the five districts (Jigjiga city, Kebribeyah town, Aubere town, Bombas town and Babile town) of the zone. Mothers, who were deaf, or critically ill, as well as mothers whose index infant had developmental delay were excluded from the study.

### Study design and data collection

The study design was a comparative cross-sectional study. Quantitative data were collected by face-to face interview technique, using pre-tested structured questionnaires, adapted from the Ethiopian Health and Demographic Survey (EDHS) 2011, and WHO [10, 25]. The questionnaire contained questions on infant characteristics (age and sex), maternal demographic characteristics (age, occupation, income, education and marital status) breastfeeding knowledge, obstetric characteristics, and EBF practices. It was initially contextualized and prepared in English and then translated into “*Somaligna*” and “*Amharic*”, and it was translated back into English to check its consistency.

Census was conducted in each study areas before the actual data collection to determine the total number of mothers of index infants of 3–5 months. A total of 583 mothers of index infants ages 3–5 months (122 employed and 461 unemployed mothers) were recruited by census. Trained Health Extension Workers and diploma nurses administered the questionnaire after obtaining informed consent from the respondents. To ensure the quality of the data, the questionnaire was pre-tested in a purposively selected town that has not been selected for the study. Data was manually checked and coded for completeness, consistency and validity by supervisors and principal investigators.

In this study, the outcome variable was the status of EBF of infants three to five months of age. EBF was defined as feeding only breast milk (including milk expressed or from a wet nurse) without anything else in the last 24 h preceding the interview except for ORS, drops, and syrups (vitamins, minerals, medicines) for therapeutic purposes [26]. Regarding the employment status of mothers, only those who were actively working full time in either governmental organization or NGOs by the time of data collection were considered as employed mothers, while mothers who were housewives (without a job) were considered as unemployed in our study. Mothers who received either institutional support, or extended maternity or leave were excluded from the study. Mothers who were working in private businesses such as merchants were also excluded from the study for two main reasons: First, there is no formal maternal leave in this type of employment. Secondly, mothers might get the chance to bring their infant with them to work site to breastfeed. The aforementioned reasons above may underestimate the effect of maternal employment on EBF practices. Additionally, early initiation of breastfeeding was defined as initiation of breastfeeding within one hour of birth of the neonate.

### Statistical analysis

Data entry was made using EPI INFO version 3.5.1 software package and analyzed using SPSS version 16. A binary logistic regression model with enter logistic regression analysis method was utilized to examine the effect of maternal employment on EBF. Variables with *p*-value of less than 0.2 in the bivariate analyses were entered multivariate logistic regression model. Crude and adjusted odds ratios are presented with a 95% confidence interval to measure the strength of association. Variables with p-value of < 0.05 were declared statistically significant. Model fitness was tested by using the Hosmer-Lemeshow goodness-of-fit and Omnibus tests of model coefficients tests with enter procedure. By using the Variance Inflation Factor (VIF) test, the Tolerance test, and values of the standard error; the explanatory variables were tested for multicollinearity before entering them into the multivariable model.

## Results

### Characteristics of the study participants

Among the total 583 mother-infant pairs recruited by census, 558 were included in the analysis. Thus, the response rate was 95.7% and the remaining 4.3% were excluded from the analysis due to incompleteness, and inconsistencies.

A total of 445 (79.7%) unemployed and 113 (20.3%) employed mothers of index infants of 3–5 months participated in the study. The mean and median ages of the mothers were 25.64 and 25.49 years old respectively for employed mothers, and 25 years old for unemployed mothers. Sixty (53.1%) of the respondents were Muslim and 160 (39.4%) were Christian among the employed mothers while 319 (71.7%) of the respondents were Muslim and 126 (28.3%) were Christian among the unemployed mothers. Majority, 88.5 and 92.1% were married in union, among the employed and unemployed mothers respectively. Regarding the educational status of the mothers; 76 (67.3%) secondary and higher, followed by primary school 19 (16.8%), informal education 14 (12.4%), and illiterate 4 (3.5%) among those employed mothers. Whereas, among the unemployed mothers; 162 (36.4%) primary school, followed by 117 (26.3%) secondary and higher, 92 (20.7%) illiterate, and 74 (16.6%) informal education. The average household income was 3632.43 (SD ± 148.45) and 2873.24 (SD ± 82.99) Ethiopian Birr per month, among the employed and unemployed mothers respectively (Table 1).

**Table 1:** Socio-demographic characteristics of the study participants

Variables	Maternal employment		
	Employed N (%)	Unemployed N (%)	*P* value*
Maternal Age			
17–19	3 (2.7)	36 (8.1)	0.049
20–24	37 (32.7)	146 (32.8)	
25–29	53 (46.9)	160 (36.0)	
≥ 30	20 (17.7)	103 (23.1)	
Maternal Education			
Illiterate	4 (3.5)	92 (20.7)	0.000
Informal education	14 (12.4)	74 (16.6)	
Primary	19 (16.8)	162 (36.4)	
Secondary and higher	76 (67.3)	117 (26.3)	
Ethnicity			
Somali	44 (38.9)	226 (50.8)	0.024
Others	69 (61.1)	219 (49.2)	
Religion			
Muslim	60 (53.1)	319 (71.7)	0.000
Christian	53 (46.9)	126 (28.3)	
Age of Infant			
3 months	21 (18.6)	126 (28.3)	0.012
4 months	30 (26.5)	142 (31.9)	
5 months	62 (54.9)	177 (39.8)	
Sex of Infant			
Male	57 (50.4)	212 (47.6)	0.594
Female	56 (49.6)	233 (52.4)	
Husband education			
Illiterate	4 (3.7)	25 (5.7)	0.569
Informal education	7 (6.4)	42 (9.6)	
Primary	16 (14.7)	65 (14.8)	
Secondary and higher	82 (75.2)	306 (69.9)	
Husband Occupation			
Farmer/Pastoralist	20 (18.3)	81 (18.5)	0.092
Daily laborer	11 (10.1)	76 (17.4)	
Government Employee	35 (32.1)	94 (21.5)	
NGO Employee	10 (9.2)	33 (7.5)	
Merchant	33 (30.3)	154 (35.2)	
Average family income (in Birr)			
500–2000	20 (18.0)	183 (43.0)	0.000
2001–3500	42 (37.8)	139 (32.6)	
3501–10000	49 (44.1)	104 (24.4)	

### Breastfeeding knowledge and obstetric characteristics

The entire employed and unemployed mothers supported the importance of breast feeding for infant’s health. Majority, 80 (70.8%) of employed and 308 (69.2%) of unemployed mothers supported the importance of breastfeeding for maternal health respectively. Almost all of the respondents, 112 (99.1%) of employed and 440 (98.9%) of unemployed mothers said breast milk is better than infant formula. Most of mothers, 90 (79.6%) of employed and 341 (76.6%) of unemployed mothers were aware of the recommended six months duration of Exclusive Breastfeeding respectively. On the other hand, 105 (92.9%) of employed and 396 (89.0%) of unemployed mothers visited the health facility at least once during their pregnancy with mean number of ANC visits of 3.9 (SD ± 0.2) and 3.9 (SD ± 0.1) respectively. Regarding to information about EBF, 154 (81%) of employed and 166 (69%) of unemployed mothers were informed about EBF respectively (Table 2).

**Table 2:** Breastfeeding knowledge and obstetric characteristics among the study participants

	Maternal Employment		
Variables	Employed N (%)	Unemployed N (%)	*P* value*
Breastfeeding is important for maternal health			
Yes	80 (70.8)	308 (69.2)	0.073
No	16 (14.2)	38 (8.5)	
Don’t know	17 (15.0)	99 (22.2)	
Infant should be put to breast immediately after birth			
Yes	104 (92.0)	395 (88.8)	0.480
No	1 (0.9)	11 (2.5)	
Don’t know	8 (7.1)	39 (8.8)	
Breast milk is better than infant formula			
Yes	112 (99.1)	440 (98.9)	0.754
No	0 (0.0)	2 (0.4)	
Don’t know	1 (0.9)	3 (0.7)	
Perceived Duration of EBF			
Six months	90 (79.6)	341 (76.6)	0.782
Other than six months	21 (18.6)	96 (21.6)	
Don’t know	2 (1.8)	8 (1.8)	
ANC attended			
Yes	105 (92.9)	396 (89.0)	0.218
No	8 (7.1)	49 (11.0)	
Place of delivery			
Health facility	101 (89.4)	397 (89.2)	0.959
Home	12 (10.6)	48 (10.8)	
Mode of delivery			
Vaginal	104 (92.0)	425 (95.5)	0.138
Caesarean section	9 (8.0)	20 (4.5)	

### Exclusive breastfeeding practices

The study revealed that the prevalence of EBF practices was 24.8and 82.9% among employed and unemployed mothers respectively in the 24 h preceding the survey. On the one hand, being engaged at work place 68 (81.9%), the perception that breast milk only is not enough 8 (9.6%), and decreased milk secretion 4 (4.8%), as well as illness of the mother 3 (3.6%) were the reasons stated by the employed mothers for feeding their infant something other than breast milk in the past 24 h. On the other hand, the perception that breast milk only is not enough 35 (45.5%), decreased milk secretion 34 (44.2%), and illness of the mother 8 (10.4%) were the reasons stated by the unemployed mothers for feeding their infant something other than breast milk in the past 24 h. Regarding to complementary feeding, 68 (60.2%) and 42 (9.4%) infant formula, 12 (10.6%) and 19 (4.3%) plain water or with additives, 21 (18.6%) and 10 (2.2%) semisolids, 21 (18.6%) and 9 (2.0%) cow milk, among employed and unemployed mothers respectively in the 24 h prior to the survey (Table 3).

**Table 3:** Exclusive breastfeeding practices among the study participants

Variables	Maternal Employment		
	Employed N (%)	Unemployed N (%)	*P* value*
Timely initiation of breastfeeding			
Yes	95 (84.1)	382 (85.8)	0.633
No	18 (15.9)	63 (14.2)	
Pre-lactal feeding			
Yes	13 (11.5)	41 (9.2)	0.462
No	100 (88.5)	404 (90.8)	
EBF status			
Exclusively Breastfed	28 (24.8)	369 (82.9)	0.000
Non-Exclusively Breastfed	85 (75.2)	76 (17.1)	
Reason for Non-exclusive Breast feeding			
Decreased milk secretion	4 (4.8)	34 (44.2)	0.000
Only Breast milk not enough	10 (9.6)	35 (45.5)	
Was at work place	68 (81.9)	0 (0.0)	
Illness of mother	3 (3.6)	8 (10.4)	

### Factors associated with EBF

In the bivariate analysis, being an unemployed mother, younger age of the infant, timely initiation of breastfeeding, having positive attitude towards breastfeeding an infant in a public place, and delivering at a health facility were significantly associated with EBF. Controlling for other variables, being an unemployed mother, monthly income, timely initiation of breastfeeding, and younger age of infant were significantly associated with EBF in the multivariate logistic regression model for the overall study participants.

Among all, those mothers who were unemployed were twenty six times more likely to practice EBF compared to those employed mothers [Adjusted OR = 26.5; 95% CI (13.6, 51.6)]; mothers with monthly income of 500–2000, and 2001–3500 birr were twice as likely to practice EBF compared to those with above 3501 birr monthly income [Adjusted OR = 2.7; 95% CI (1.4, 5.2)]; and [Adjusted OR = 2.2; 95% CI (1.2, 4.0)] respectively; mothers who completed secondary and higher education were about four times more likely to practice EBF compared to those who were illiterate [Adjusted OR = 3.8; 95% CI (1.5, 9.5)]; those who initiated breastfeeding within one hour of delivery were about three times more likely to practice EBF compared to their counterparts [Adjusted OR = 2.6; 95% CI (1.4, 4.8)]; mothers of 3 months and 4 months age infants were about three times more likely to practice EBF compared to those mothers of 5 months age infants [Adjusted OR = 2.2; 95% CI (1.2, 4.1)], and [Adjusted OR = 2.2; 95% CI (1.2, 3.8)] respectively. The other variables were not significantly associated with EBF practice after adjusting for confounders (Table 4).

**Table 4:** Factors associated with exclusive breastfeeding practices

Variables	Non-EBF	EBF	Crude OR (95% CI)	Adj. OR (95% CI)
Maternal Employment				
Employed	85	28	1	1
Unemployed	76	369	14.7 (9.0, 24.1)**	**26.5 (13.6, 51.6**)**
Maternal Education				
Illiterate	27	69	1	1
Informal education	28	60	0.8 (0.5, 1.6)	1.1 (0.5, 2.4)
Primary	39	142	1.4 (0.8, 2.5)	1.9 (0.9, 3.9)
Secondary and higher	67	126	0.7 (0.4, 1.3)	3.8 (1.5, 9.5)*
Age of Infant				
3 months	27	120	3.1 (1.9, 5.1)**	2.2 (1.2, 4.1)*
4 months	36	136	2.6 (1.7, 4.1)**	2.2 (1.2, 3.8)*
5 months	98	141	1	1
An infant should be put to breast immediately after birth?				
Yes	139	360	1.5 (0.9, 2.7)	1.4 (0.7, 3.0)
No	22	37	1	1
Place of delivery				
Health facility	137	361	1.8 (1.0, 3.1)	1.0 (0.5, 2.2)
Home	24	36	1	1
Timely initiation of breast feeding				
Yes	123	354	2.5 (1.6, 4.1)**	2.6 (1.4, 4.8)*
No	38	43	1	1
Mother received information/advice on BF				
Yes	85	204	1	1
No	76	193	0.9 (0.7, 1.4)	1.4 (0.8, 2.5)
History of breast disease				
Yes	26	42	1	1
No	135	355	1.6 (0.9, 2.8)	1.9 (0.9, 3.6)
Breastfeeding at public place is wrong				
True	88	167	1	1
False	73	230	1.7 (1.2, 2.4)*	1.5 (0.9, 2.6)
Husband support				
Yes	113	252	0.7 (0.4, 1.0)	0.7 (0.4, 1.2)
No	42	140	1	1
Average family income (in Birr)				
500–2000	41	162	4.3 (2.4, 7.7)**	2.7 (1.4, 5.2)*
2001–3500	51	130	1.6 (0.9, 2.5)	2.2 (1.2, 4.0)*
3501–10000	65	88	1	1

*- Significance level of < 0.05 **- Significance level of < 0.001

## Discussion

This study provides information regarding the effect of maternal employment on EBF among mothers of index infants of 3–5 months, living in the Semi-pastoralist community of Eastern border of Ethiopia where many governmental and non-governmental organizations are located. This study has demonstrated a prevalence of 24.8 and 82.9% of EBF practices among employed and unemployed mothers of index infants of 3–5 months respectively in the 24 h preceding the survey. Being an unemployed mother, timely initiation of breastfeeding, and younger age of infant were significantly associated with EBF.

The prevalence of EBF in our study was low among employed and high among unemployed mothers, which is in line with studies conducted in different parts of Ethiopia [7, 8, 16]. In a similar trade, unemployed mothers were twenty-five times more likely to exclusively breastfeed their infants as compared to those employed mothers. This indicates that maternal employment is a hindrance for EBF. In agreement with this, studies from Ethiopia [7, 8, 16, 17, 27], peninsular Malaysia [18, 28], Saudi Arabia [29, 30], and Canada [31] reported that maternal employment has a negative effect on EBF. Many reasons can be stated for this association. In accordance with the Constitution of Ethiopia and Labor Proclamation, female workers are entitled to fully paid maternity leave of 90 days (30 days antenatal and 60 days postnatal) on recommendation of medical doctor. Hence employed mothers will have a maximum of two or three months to stay at home and breastfed their infants which doesn’t fit with the recommended six months of EBF. This short maternity leave period successively influences them to introduce complementary feeding starting from the time they return to work [21, 32]. Another study [33] reported that, each week of maternity leave increased the duration of breastfeeding by almost one-half week. Besides, since there is no breastfeeding friendly environment like facilities for breastfeeding at workplaces, employed mothers couldn’t take their infants to the workplace and breastfeed there [21, 34]. Therefore, the summative effect of the stated reasons above, would compromise the rate of EBF among employed mothers. To the contrary, some other studies [35, 36] have reported that maternal employment has no effect on EBF.

In this study, mothers with a lower monthly household income had high tendency to practice EBF. This agrees with findings from Ethiopia [37] and South Africa [38]. This could be explained by the reason that breastfeeding is the only option for mothers with lower monthly income as they cannot afford to buy other alternative foods. Nevertheless, it is difficult to conclude since monthly household income is not enough of an indicator of socioeconomic status; rather wealth index should have been done to determine this variable. Hence, this can be quoted as a limitation of the present study. On the other hand, mothers whose educational status was secondary or higher were more likely to practice EBF in the present study. This is in line with the research findings from Ethiopia [35], United Arab Emirates [39], and India [40]. The possible explanation for this association could be the fact that educated mothers may have a better knowledge regarding child feeding practices as they are prone to get information from different channels. Early initiation of breastfeeding was positively associated with EBF practice. This is in accordance with findings from Ethiopia, China and Egypt [9, 16, 35, 41, 42]. This could be due to the fact that early suckling stimulates the release of prolactin, which helps in the production of milk, and is responsible for the ejection of milk [3, 9, 10]. Hence, if there is delayed initiation of breastfeeding, the production of breast milk would be inhibited due to limited stimulation of the neonate to suckle, as a result to the initiation of pre-lactal foods.

This study revealed that child’s age had significant association with EBF practice. Infants in age group of 3 and 4 months were three times more likely to receive EBF as compared to those whose age was 5 months. This finding was consistent with the studies conducted in other parts of Ethiopia [7, 8, 35, 43], Nigeria [44], and Uganda [45]***.*** Different studies indicate that the rate of EBF decreases significantly as the age of infants come close to 6 months in Iran, Uganda, Sudan, and Ethiopia [8, 43, 45–47]. The first possible reason for this association could be the traditional postpartum rest in the first few months after delivery that obligates women to stay at home. This, as a result may encourage them to breastfeed their infant. The other explanation may be due to the perception that some mothers have, that breast milk alone is not enough as the infant gets older [7, 8].

The present study has its own strengths and limitations. We only included mothers of index infants of 3–5 months to determine the real effect of maternal employment on EBF practices, as employed mothers with infants aged less than 3 months couldn’t be hindered by their employment since they would be on maternity leave. The use of comparison group in our study has boosted its ability to draw inferential causalities between the independent variables and the practice of EBF. In ascertaining EBF, we used the 24-h recall method to determine EBF which currently is recommended by WHO. Hence, we minimized the recall bias in ascertaining EBF that could have occurred by the other methods. Nevertheless, there are some limitations inherent to this method. As per the report of many studies [48–52], since the single 24 h recall only captures a single glance, it may not represent the usual intake of an infant if there is a day to day variation in the feeding pattern, which may consequently lead to an overestimation and misclassification. In addition, there might be a recall bias in ascertaining some variables like early initiation of breastfeeding and other maternal health care related variables. We included only government and NGO full time workers since there is no formal maternity leave in the other employment types such as traders. For that reason, this study doesn’t represent all employed mothers.

## Conclusions

In conclusion, the prevalence of EBF practices in the study area was very low among mothers employed in governmental and NGOs but high among unemployed mothers, indicating a significant difference between the two groups. Hence, maternal employment in governmental organizations or NGOs may have a negative influence on EBF practices. In addition, early initiation of breastfeeding, low income, maternal education and younger infant age, were also significantly associated with EBF practice. In developing countries like Ethiopia where the rate of urbanization increases through time, the tendency of women to be educated and employed would be high. In effect, this reduces the chance of their infants to be exclusively breastfed, and in turn increases under-five mortality due to infant malnutrition. Thus, we recommend that breastfeeding-friendly work environment should be launched for working mothers by establishing work-site day care centers for infants in order to promote of EBF. Moreover, Information, Education and Communication (IEC) programs on EBF practices should be provided mainly for employed mothers with a special focus on timely initiation of breastfeeding. Further analytical studies, especially follow-up studies, in big cities of the country where there is a high proportion of employed mothers is suggested to explore the effect of maternal employment on EBF practices.

## Data Availability

The datasets generated during and/or analyzed during the current study are available from the corresponding author on reasonable request.

## Abbreviations

EBF: Exclusive Breastfeeding
EDHS: Ethiopian Health and Demographic Survey
IEC: Information, Education and Communication
NGO: Non-Governmental Organization
ORS: Oral Rehydration Salt
SDG 2: Sustainable Development Goal 2
WHO: World Health Organization
